# Personality traits and the risk of becoming lonely in old age: A 5-year follow-up study

**DOI:** 10.1186/s12955-020-01303-5

**Published:** 2020-02-28

**Authors:** Heidi Ormstad, Grethe Eilertsen, Trond Heir, Leiv Sandvik

**Affiliations:** 1 Faculty of Health and Social Sciences, University of South-Eastern Norway, P.O. Box 7053, NO-3007 Drammen, Norway; 2 Norwegian Center for Violence and Traumatic Stress Studies, Oslo, Norway; 3 Institute of Clinical Medicine, University of Oslo, Oslo, Norway

**Keywords:** Gender perspective, Loneliness, Longitudinal study, Personality traits

## Abstract

**Background:**

Although many people experience loneliness in old age, there is little knowledge of predisposing personality factors. The aim of the present study was to explore to what extent personality traits are associated with the risk of becoming lonely, in women and men aged 60–79 years at baseline.

**Methods:**

The panel data are from The Norwegian study on Life course, Ageing and Generations (NorLAG)**.** Our sample consisted of 516 men and 419 women aged 60–79 years, who were surveyed in both 2002–2003 (baseline) and 2007–2008 (follow-up), and who reported not being lonely at baseline. Personality traits were measured by the Big Five scale. Multivariable logistic regression analyses were used to investigate the association between a personality trait and the risk of becoming lonely, with adjustment for age, mental health and living with a partner.

**Results:**

At follow-up 59 women and 54 men reported loneliness (14.1% vs. 10.5%, *p* = 0.092). Among women, high agreeableness at baseline was significantly associated with a higher risk of becoming lonely. Among men, low agreeableness, low conscientiousness and high neuroticism at baseline were significantly associated with a higher risk of becoming lonely.

**Conclusions:**

Personality traits related differently to loneliness depending on gender. These findings may be useful when developing strategies for preventing loneliness in old age.

## Background

Loneliness and isolation are parts of the experience of growing old [1]. Due to exposure to age-related changes and losses, older persons are particularly vulnerable to loneliness [2]. Examples of age-related changes and losses are the loss of a partner and friends through death, worsening health, and loss of social roles through retirement [2].

Reported prevalence of loneliness among the elderly range from 39 to 72% [3–7]. The considerable variation in these estimates may partly be caused by the absence of a universally accepted definition of loneliness. Thus, a range of indicators and measurement tools of loneliness are used.

Several studies have shown that loneliness in old age is strongly associated with depression, and that both loneliness and depression have serious negative effects on well-being [6, 8–11]. Further, both loneliness and depression are risk factors for early death [12, 13]. In a recent study by Holwerda et al., it was shown that loneliness and depression are important predictors of early death in older adults, and that severe depression is strongly associated with excess mortality in older men who were lonely [14]. Furthermore, they found that the combination of either emotional or social loneliness with severe depression is a lethal combination in men in the long term. Thus, health authorities should develop interventions aimed at reducing the prevalence of loneliness in old age. In this context, increased knowledge about causes of loneliness may be helpful.

Several studies have aimed to explore factors associated with loneliness in old age. In a recent review by Cohen-Mansfield et al. [15], in which 38 mainly cross-sectional studies were reviewed, the variables significantly associated with loneliness in older adults were: female gender, non-married status, older age, low income, lower educational level, living alone, low quality of social relationships, poor self-reported health, and poor functional status. Further, psychological attributes associated with loneliness included poor mental health, low self-efficacy beliefs, negative life events, and cognitive deficits.

A few studies have addressed the role of personality traits when experiencing loneliness in old age. Hensley et al. studied participants from the Georgia Centenarian Study, and found that both extraversion and neuroticism significantly predicted loneliness [16]. Bishop and Martin [17] also found that neuroticism directly affected loneliness, and further, that educational attainment indirectly affected loneliness via neuroticism. Long and Martin (2000) reported that neuroticism was positively associated with loneliness in the oldest old [18]. As far as we can see, none of the above-mentioned studies investigated women and men separately, and none of them had a longitudinal design. Thus, more research is needed on the association between personality traits and loneliness in old age, applying a gender perspective. Moreover, studies with a longitudinal design are requested [1], since they will enable an improved understanding of causal order.

Over the past 40 years, a number of surveys have shown that personality traits tend to spread over five dimensions, the so-called ‘Big Five’ [19], including the following five traits; extraversion (dominance, extraversion, outgoing), agreeableness (human friendliness, warmth), conscientiousness, neuroticism (anxious, negative emotions), and openness to experience (openness, openness to impressions).

Based on growing evidence concerning the detrimental aspects of loneliness, we aim to explore to what extent the five personality traits in the Big Five are associated with the risk of becoming lonely in old age, focusing on a gender perspective.

The aim of the present study was to explore to what extent personality traits are associated with becoming lonely, based on self-reported loneliness among women and men aged 60–79 years at baseline.

## Methods

The present study is based on data from the Norwegian study of life course, ageing and generations, NorLag [20]. This is a longitudinal panel study of Norwegian individuals in mid-life and old age. The panel design of the study offers the possibility to explore the premises for vital aging and wellbeing in old age, and to contribute knowledge to a sustainable welfare policy in an aging society. The database from the study includes data from variables measuring loneliness, personality traits measured by the Big Five scale, and variables associated with loneliness.

Our sample consists of 516 men and 419 women who were surveyed in both 2002–2003 (T1) and 2007–2008 (T2), aged 60–79 years at T1, and did not report loneliness at T1. Personality traits were measured by the Big Five scale.

### The big five

Several studies the last 40 years have shown that personality traits tend to distribute along five dimensions, called ‘The Big Five’ [21]. These dimensions are called “extraversion”, “agreeableness”, “conscientiousness”, “neurotism” and “openness to experience”.

In the NorLag study, a 20 items version of the Big Five scale was used [22]. These Big Five data were used in our study when studying the associations between personality traits and the risk of becoming lonely.

### Loneliness

The NorLag study includes data on three questions regarding loneliness, recorded at both baseline and follow-up. The number of missing data differed markedly between these questions. We decided to base our definition of loneliness on the question ‘have you felt lonely during the last week?’, because the number of missing data was much lower for this question than for the other loneliness questions. This was thus used as ***dependent variable.*** Possible answers to this question were ‘never’, ‘seldom’, ‘sometimes’ and ‘often’. We defined that a person was lonely if he answered ‘sometimes’ or ‘often’ to this question. Thus, the dependent variable in our study is whether the person felt lonely at follow-up.

The following baseline variables were chosen to be ***independent variables*** in the present study: *Big Five* [22], *age, gender, living with a partner* (yes/no), *SF-12 mental health* (Short form 12 health survey) [23, 24], *CES-D* (Center for Epidemiologic Studies Depression scale) [25] and *HSCL anxiety* [26, 27].

#### Statistical analysis

A chi-squares test was used when comparing frequencies in two groups. Multivariable logistic regression analyses were used to investigate the associations between personality traits and the risk of becoming lonely, with adjustment for the baseline variables age, SF-12, CES-D, HSCL anxiety and living with a partner. The results are presented as odds ratios with 95% confidence intervals and *p*-values. The assumptions underlying logistic regression analysis were checked, and found to be adequately met in each regression model. A significance level of 5% was used. The statistical analysis was performed by using IBM-SPSS version 22.

## Results

Our sample included 516 men and 419 women above 60 years, who reported not being lonely at baseline. Five years later, 54 (10.5%) of the men and 59 (14.1%) of the women reported that they felt lonely (*p* = 0.092). The basic variables are presented, separately for women and men, in Table 1.

**Table 1:** Description of the variables

Variable	Women	N	Men	N	p-value
Age, mean	67.7 ± 5.4	419	67.6 ± 5.4	516	0.826
Agreeableness, mean	23.7 ± 3.4	344	21.9 ± 3.6	424	**< 0.001**
Extraversion	18.6 ± 4.0	350	18.0 ± 3.7	427	0.060
Conscientiousness	20.4 ± 4.1	329	20.5 ± 3.5	424	0.940
Neuroticism	12.9 ± 5.0	348	11.4 ± 4.4	426	**< 0.001**
Openness to experience	19.5 ± 3.6	347	19.6 ± 3.1	427	0.521
SF-12 mental health	56.7 ± 6.6	419	57.8 ± 5.5	514	**0.013**
CESD depression scale	9.2 ± 6.3	330	8.7 ± 6.1	403	0.305
HSCL anxiety	1.18 ± 0.26	353	1.13 ± 0.23	429	**0.003**
Living with a partner	263 (62.8%)	419	434 (84.1) %	516	**< 0.001**

Associations between personality traits and the risk of becoming lonely, after adjustment for the baseline variables age, SF-12, CES-D, HSCL anxiety and living with a partner, are investigated separately for women and men, and the results are presented in Tables 2 and 3.

**Table 2:** Big Five personality traits as predictors of becoming lonely, for women*

Personality trait		OR	95% CI	p-value
Agreeableness	Tertile 2 vs. tertile 1	1.03	0.40–2.62	0.957
	Tertile 3 vs. tertile 1	2.74	1.21–6.18	**0.015**
Extraversion	Tertile 2 vs. tertile 1	1.37	0.67–2.81	0.390
	Tertile 3 vs. tertile 1	0.87	0.36–2.07	0.749
Conscientiousness	Tertile 2 vs. tertile 1	0.62	0.25–1.55	0.307
	Tertile 3 vs. tertile 1	0.83	0.38–1.80	0.632
Neuroticism	Tertile 2 vs. tertile 1	1.23	0.56–2.69	0.615
	Tertile 3 vs. tertile 1	1.10	0.48–2.56	0.820
Openness to experience	Tertile 2 vs. tertile 1	0.89	0.40–1.95	0.763
	Tertile 3 vs. tertile 1	1.34	0.65–2.93	0.407

*Adjusted for age, mental health (SF-12), and living with partner at T1

**Table 3:** Big Five personality traits as predictors of becoming lonely, for men*

Personality trait		OR	95% CI	p-value
Agreeableness	Tertile 2 vs. tertile 1	0.54	0.25–1.17	0.118
	Tertile 3 vs. tertile 1	0.34	0.14–0.80	**0.014**
Extraversion	Tertile 2 vs. tertile 1	0.85	0.41–1.77	0.665
	Tertile 3 vs. tertile 1	0.73	0.30–1.74	0.472
Conscientiousness	Tertile 2 vs. tertile 1	0.53	0.25–1.14	0.102
	Tertile 3 vs. tertile 1	0.31	0.12–0.76	**0.010**
Neuroticism	Tertile 2 vs. tertile 1	1.61	0.61–4.21	0.334
	Tertile 3 vs. tertile 1	3.55	1.45–8.67	**0.005**
Openness to experience	Tertile 2 vs. tertile 1	0.57	0.25–1.27	0.170
	Tertile 3 vs. tertile 1	0.77	0.34–1.74	0.536

*Adjusted for age, mental health (SF-12), and living with partner at T1

High agreeableness was associated with a higher risk of becoming lonely in women. For men, however, high agreeableness was associated with a lower risk of becoming lonely. Also, conscientiousness was associated with a lower risk of becoming lonely in men, but not in women. Furthermore, neuroticism was associated with a higher risk of becoming lonely in men, but not in women.

In the first, second and third agreeableness tertile the percentage of women becoming lonely was 9.0, 8.6 and 19.7%, respectively.

In the first, second and third agreeableness tertile the percentage of men becoming lonely was 17.2, 8.6 and 5.7%, respectively. Corresponding results for neuroticism was 5.0, 7.9 and 20.5%, and corresponding results for conscientiousness was 15.4, 9.3 and 5.6%.

## Discussion

In order to explore longitudinal associations between personality traits and the risk of becoming lonely, we based our study on a representative sample of elderly people in Norway. We included participants who did not report loneliness at baseline. In this sample, 14.1% of the women and 10.5% of the men felt lonely 5 years later. Personality traits related differently to loneliness depending on gender. Among women, loneliness was associated with higher levels of agreeableness. Among men, loneliness was associated with lower levels of agreeableness, lower levels of conscientiousness, and higher levels of neuroticism.

Our findings that neurotic men became lonely more often than other men, is in accordance with gender-unspecific findings from populations of the oldest old [16, 18]. As far as we can see, no findings have been reported about the association between loneliness and agreeableness or, conscientiousness, the other two personality traits showing associations in our study. There may be several possible explanations for the associations between personality traits and loneliness. Firstly, personality traits may influence people’s ability to create or maintain friendships, family relationships or well-functioning social networks. Thus, men that are less agreeable, less conscientious, or more neurotic, may have less social contact simply because they have a lesser ability to establish and maintain social relationships. In this regard, it has, interestingly, been reported that for men, but not for women, a low level of social contacts and reduction of social contacts predicted loneliness [28].

Secondly, personality may affect people’s emotional state, including a sense of loneliness that is independent of actual social interaction. For example, women with elevated levels of agreeableness may miss people to care for, and thus feel lonely, although they are not socially isolated. This interpretation is supported by a qualitative study reporting elderly describing agonizing loneliness together with feeling less valuable [29]. In particular women expressed feeling bitter about no longer being important enough in the family, or feeling redundant and not interesting. Moreover, it has been reported that women living with a partner are more likely than men to experience children, family, and friends as sources of support [30]. Older women in Western countries seem to represent a generation in which traditional female roles were strongly tied to the home and family [31]. A loss of these roles may induce a feeling of loneliness, and probably more agreeable women are particularly exposed.

Concerning methodological considerations, it is important to realize that loneliness is related to but not equivalent to social isolation. People can be alone without feeling lonely, or experience loneliness in social settings. Data on the availability and use of different social networks would have made it easier to interpret relationships with personality traits. Further, we do not know the level of loneliness among non-responders. Thus, response bias may have affected the estimated prevalence of loneliness in the population. However, we believe that a potential response bias may primarily affect the frequency estimates of loneliness or personality traits and to a lesser extent their relationship [32, 33]. The main strength of the present study is the longitudinal design with the gender perspective.

## Conclusions

Our study suggests that some personality traits are associated with the risk of becoming lonely in old age. Further, these associations differed markedly between men and women.

Loneliness is an unpleasant emotional state that is associated with lack of social integration. Its connection to increased risk of disease [15, 34, 35] or early death [36] emphasizes the importance of measures to counter loneliness in the elderly. For the aging population leaving work, it is important to have other gathering places that can strengthen connectedness and social interaction. Personality consists of relatively stable personality traits that is difficult to change. However, knowing that certain personality traits are related to loneliness later in life may increase the awareness of maintaining social relationships into old age.

## Data Availability

The NorLAG data are distributed by the Norwegian Social Science Data Services. Interested researchers can contact project leader Heidi Ormstad (heidi.ormstad@usn.no) with a request for the particular data set used in the present study.

## Abbreviations

NorLAG: The Norwegian study on Life course, Ageing and Generations
